# A revolution in health: Opportunities and challenges of the Metaverse

**DOI:** 10.17179/excli2022-5017

**Published:** 2022-05-31

**Authors:** Milad Ahmadi Marzaleh, Mahmoudreza Peyravi, Fatemeh Shaygani

**Affiliations:** 1 Department of Health in Disasters and Emergencies, Health Human Resources Research Center, School of Management and Medical Informatics, Shiraz University of Medical Sciences, Shiraz, Iran; 2 Student Research Committee, Shiraz University of Medical Sciences, Shiraz, Iran

## ⁯⁯

Nowadays, technology has changed the way individuals interact with the actual world around. Since 2021, Metaverse has received a lot of attention globally and has been the hot topic of the technology world (Clark, 2021[2]). This term refers to the shared online space integrating the virtual and the real worlds (physical, augmented, and virtual reality), which people can enter using digital identities. It gives each user an underlying environment, presenting a consistent state to all users where they can have their perspective on the virtual world. This ternary digital world has been widely debated with the fast digital economy growth and has been considered the infrastructure for the next generation Internet (Kim, 2021[3]). In the past few years, various technologies such as artificial intelligence, big data, cloud computing, telemedicine, blockchain, and virtual and augmented reality have changed the healthcare industry. For instance, Facebook's rebranding as Meta in October 2021 has led several well-known technology companies to start and accelerate their business models on the basis of the Metaverse, and the healthcare sector is no exception (Lee, 2021[4]; Chen and Zhang, 2022[1]). Some healthcare startups and companies have also started moving to the Metaverse to use this technology for providing healthcare services. This letter has been written to emphasize the importance of Metaverse and its following opportunities and challenges in the healthcare field in 2022.

Futurists and technologists have assessed how healthcare services may be changed and promoted by the Metaverse in future. Within the recent pandemic, augmented reality has been used by the World Health Organization (WHO) to train COVID-19 respondents and mental health experts how to utilize the virtual reality for treating people with mental illnesses and emotional difficulties such as post-traumatic stress, phobias, anxiety disorders, hallucinations, and delusions. It has also been used in medical schools for educational purposes. Thus, the Metaverse seems to be beneficial in helping medical professionals in many aspects of their work involving the use of these technologies.

Currently, surgeons working in leading hospitals and universities use technologies such as augmented reality, virtual reality, and artificial intelligence. Although these technologies provide a 3D view of a patient's body and help surgeons plan and perform surgeries more efficiently, they have several disadvantages such as creating realistic surgical objects within a computer-generated space, providing pocket size immersion with a limited resolution, and restriction to specific clinical settings. Metaverse can overcome this challenges via providing realistic interaction amongst doctors, patients, and objects. Other benefits of Metaverse include personalized health data monitoring, analysis of patients' clinical data, and complete elimination of physical and paper-based patients' records. On the other hand, there are some challenges associated with the application of Metaverse in healthcare services including the possibility of users' privacy loss, high cost of this technology, disagreement between various health-related organizations and institutions for launching this technology, possibility of violating ethical issues in the use of the technology, possibility of threatening human health, high rate of depression, violence, and self-harm, possibility of having negative effects on the cultural security of countries and consequently affecting the health status, deepening gaps and inequalities between developed and developing countries, and possibility of going beyond the dark web and its inappropriate applications such as human trafficking for the removal of organs and body parts.

Given that health is one of the most important issues globally, governments and technology giants should fully clarify various aspects and potentials of Metaverse before its widespread use. In this context, it is necessary to make the most of its capabilities and minimize its associated concerns.

## Conflict of interest

The authors have no conflict of interest to declare.
